# Трудности дифференциальной диагностики функциональной гипоталамической аменореи и синдрома поликистозных яичников: систематический обзор

**DOI:** 10.14341/probl13529

**Published:** 2024-11-13

**Authors:** Ю. С. Абсатарова, Ю. С. Евсеева, Е. Н. Андреева, Е. В. Шереметьева, О. Р. Григорян, Р. К. Михеев

**Affiliations:** Национальный медицинский исследовательский центр эндокринологии; Национальный медицинский исследовательский центр эндокринологии; Национальный медицинский исследовательский центр эндокринологии; Российский университет медицины; Национальный медицинский исследовательский центр эндокринологии; Национальный медицинский исследовательский центр эндокринологии; Национальный медицинский исследовательский центр эндокринологии

**Keywords:** функциональная гипоталамическая аменорея, синдром поликистозных яичников, гиперандрогения, нервная анорексия, ановуляция

## Abstract

**ВВЕДЕНИЕ:**

ВВЕДЕНИЕ. Функциональная гипоталамическая аменорея (ФГА) и синдром поликистозных яичников (СПЯ) — это патологии, наиболее часто встречающиеся у женщин в репродуктивном возрасте. ФГА развивается на фоне психоэмоционального стресса или чрезмерных физических нагрузок и характеризуется угнетением нейроэндокринной регуляции оси гипоталамус-гипофиз-яичники с последующим снижением выработки половых стероидов. Для СПЯ важнейшими патогенетическими звеньями являются инсулинорезистентность и гиперандрогения. Патология нейроэндокринной регуляции при овариальной гиперандрогении сопровождается избыточной пульсирующей секрецией гонадотропин-рилизинг-гормон (ГнРГ), способствуя усилению выработки лютеинизирующего гормона (ЛГ). Как ФГА, так и СПЯ, приводит к множественным осложнениям со стороны других органов и систем: сердечно-сосудистой патологии, снижению минеральной плотности костной ткани (МПКТ) при длительной аменорее и способствует развитию бесплодия.

**ЦЕЛЬ ИССЛЕДОВАНИЯ:**

ЦЕЛЬ ИССЛЕДОВАНИЯ: изучить и проанализировать работы, изучающие проблемы дифференциальной диагностики ФГА и СПЯ.

**МЕТОДЫ:**

МЕТОДЫ. С помощью поисковых систем PubMed, eLibrary, CyberLeninka.ru проведен систематический литературный поиск статей за последние 6 лет, отвечающих следующим критериям: работы, в которых описаны методы и разработаны критерии дифференциальной диагностики ФГА и СПЯ. Также в обзор включены отдельные значимые публикации за период с 1998 по 2018 гг.

**РЕЗУЛЬТАТЫ:**

РЕЗУЛЬТАТЫ. В данном обзоре освещены дифференциально-диагностические критерии ФГА и СПЯ. Описаны особенности клинических, лабораторных и инструментальных исследований. Проанализированы публикации, описывающие сосуществование данных патологий у женщин, и подробно описаны методы, позволяющие дифференцировать эти нозологии.

**ЗАКЛЮЧЕНИЕ:**

ЗАКЛЮЧЕНИЕ. Правильно и своевременно установленный диагноз способствует назначению соответствующих схем лечения, снижает частоту развития осложнений и повышает качество жизни. В свете последних достижений в описании механизмов нейроэндокринной регуляции репродуктивной системы необходимо проводить исследования, посвященные изучению роли нейропептидов в развитии ФГА и СПЯ, что может послужить созданию более точных диагностических маркеров заболеваний.

## Abstract (en)

**BACKGROUND:**

BACKGROUND. Functional hypothalamic amenorrhea (FHA) and polycystic ovary syndrome (PCOS) are pathologies most common in women of reproductive age. Menstrual irregularities (oligo/amenorrhea) are the most common symptom of these diseases. FHA develops against the background of stress or excessive physical exertion, and is characterized by inhibition of neuroendocrine regulation of the hypothalamic-pituitary-ovarian axis with a subsequent decrease in the production of sex steroids. For PCOS, the most important pathogenetic links are insulin resistance and hyperandrogenism. The pathology of neuroendocrine regulation in ovarian hyperandrogenism is accompanied by excessive pulsatile secretion of gonadotropin-releasing hormone (GnRH), promoting increased production of luteinizing hormone (LH). Both FHA and PCOS lead to multiple complications from other organs and systems: cardiovascular pathology, decreased bone mineral density with prolonged amenorrhea and contribute to the development of infertility.

**OBJECTIVE:**

OBJECTIVE. To analyze the works studying the problems of differential diagnosis of FHA and PCOS.

**METHODS:**

METHODS. Using PubMed, eLibrary, CyberLeninka.ru, a systematic search was conducted for articles published over the past 6 years that met the following criteria: the research that describe methods and develop criteria for the differential diagnosis of FHA and PCOS. Selected impactive publications within 1998–2018 were also included in the review.

**RESULTS:**

RESULTS. This review highlights the differential diagnostic criteria for FHA and PCOS. The features of clinical, laboratory and instrumental studies are also described. Publications describing the coexistence of these pathologies in women are analyzed, and methods that allow differentiating these nosologies are described in detail.

**CONCLUSION:**

CONCLUSION. A correct and timely diagnosis facilitates the prescription of appropriate treatment regimens, reduces the incidence of complications and improves the quality of life of women. In light of recent advances in the description of the mechanisms of neuroendocrine regulation of the reproductive system, it is necessary to further conduct research to study the role of neuropeptides in the development of FHA and PCOS, which may serve to create more accurate diagnostic markers of diseases.

## ВВЕДЕНИЕ

Нарушения менструального цикла являются наиболее частым проявлением ФГА и СПЯ. Несмотря на различную этиологию и патогенез, дифференциальная диагностика этих заболеваний нередко вызывает трудности. Более серьезную проблему представляет собой сочетание данных состояний, что осложняет не только диагностику, но и выбор терапевтического вмешательства [1].

ФГА — расстройство репродуктивной функции, проявляющееся аменореей и снижением продукции эстрогенов. Особенность патологии отражена в ее названии, т.е. отсутствует органическая составляющая, и она обратима, исчезает после нормализации энергообеспечения или разрешения эмоционального стресса [2].

Диагноз «СПЯ» ставится на основании наличия двух из трех основных критериев, выделенных Европейским обществом репродукции и эмбриологии человека (ESHRE) и Американским обществом репродуктивной медицины (ASRM) (Роттердам, 2003 г.):

Стоит отметить, что СПЯ является диагнозом исключения, т.е. должны быть исключены другие эндокринопатии, сопровождающиеся нарушениями менструального цикла и гиперандрогенией.

Известно, что существует ряд нозологий, для которых характерны вторичная аменорея, гиперандрогения, снижение продукции эстрогенов. Нарушения менструального цикла и избыточная секреция мужских половых гормонов характерны для врожденной дисфункции коры надпочечников, синдрома Кушинга, андроген-продуцирующих опухолей. Гипоэстрогения и аменорея в репродуктивном возрасте — ключевые симптомы преждевременной недостаточности яичников. Гиперпролактинемия различного генеза непосредственно приводит к нарушению репродуктивной системы. Данные патологии отличны и по этиологии, и по клинической картине. В настоящее время лабораторная и инструментальная виды диагностики позволяют врачу-клиницисту подтвердить или исключить эти диагнозы. Учитывая масштабность и обширность причин нарушений менструального цикла, настоящая публикация сфокусирована на наиболее частых, спорных и не всегда однозначных заболеваниях.

Цель исследования: анализ научных и клинических исследований, посвященных проблемам дифференциальной диагностики ФГА и СПЯ, с выделением ключевых моментов, по которым будет легче дифференцировать эти заболевания, что позволит врачам-клиницистам быстро и правильно поставить нужный диагноз и назначить соответствующую терапию.

## МЕТОДЫ

## Дизайн исследования

Систематический обзор был представлен в соответствии с руководящими принципами предпочтительных элементов отчетности для систематических обзоров и метаанализов (PRISMA) [4].

Критерии приемлемости: оценка исследований на соответствие критериям включения проводилась в три этапа, таких как оценка заголовка, аннотации, полного текста статьи.

Критерии включения: все исследования (экспериментальные и наблюдательные), освещающие проблемы дифференциальной диагностики ФГА и СПЯ.

Критерии исключения: исследования, в которых диагноз «СПЯ» выставлялся путем самоанкетирования или в результате опросов.

Источники информации: два исследователя независимо друг от друга осуществляли поиск статей, опубликованных в период с 2018 по 2024 гг. в отечественных (eLibrary, CyberLeninka.ru) и международной (PubMed) базах данных. Преимущественным являлся свободный доступ к полному тексту публикаций. Также в обзор включены отдельные значимые публикации за период с 1998 по 2018 гг.

Стратегия поиска: поисковый запрос включал следующие слова: «functional hypothalamic amenorrhea»; «polycystic ovary syndrome»; «differential diagnosis»; «anovulation»; «hyperandrogenism»; «eating disorder»; «anorexia nervosa»; «infertility». В русскоязычных базах использовали термины для поиска: «функциональная гипоталамическая аменорея»; «синдром поликистозных яичников»; «дифференциальная диагностика»; «ановуляция»; «гиперандрогения»; «нарушения пищевого поведения»; «нервная анорексия»; «бесплодие».

Отбор исследований: исходно проводился скрининг названия и/или резюме потенциальных исследований, далее изучался полный текст статьи.

Процесс сбора данных: процедура отбора публикаций включала анализ на предмет соответствия ключевым словам проводимого нами исследования. Контент-анализ основывался на отборе научных исследований, соответствующих теме «дифференциальная диагностика ФГА и СПЯ».

Данные и обобщенная величина эффекта: исследование проводилось с помощью методов контент-анализа и аналитического обобщения. Выполнен анализ данных на предмет критериев постановки диагнозов ФГА и СПЯ и сложностей, возникающих при дифференциальной диагностике этих нозологий.

Извлечение данных и оценка качества: анализируемые публикации были отобраны согласно критериям включения и исключения в соответствии с целью исследования и заранее определенными условиями. Особое внимание уделялось данным о новых способах диагностики ФГА и СПЯ. В ходе исследования оценивалось качество публикаций, соответствующих теме работы, касательно включенных выборок пациенток, критериев постановки диагноза, проведенных лабораторных и инструментальных методов диагностики.

Статистический анализ: не проводился.

Синтез результатов: не проводился.

Риск предвзятости в отдельных исследованиях: не оценивался.

Дополнительные анализы: дополнительных анализов для данного исследования не предусмотрено.

## РЕЗУЛЬТАТЫ

## Отбор исследований

Проводился поиск работ, опубликованных в период с января 2018-го по январь 2024 гг., соответствующих критериям включения. Схема поиска представлена на рисунке 1. В ходе поиска, после удаления дублирующих публикаций, выявили 2373 релевантных исследования, из них отобрано 33 полнотекстовых статьи, которые отвечали критериям приемлемости для данной работы.

**Figure:**
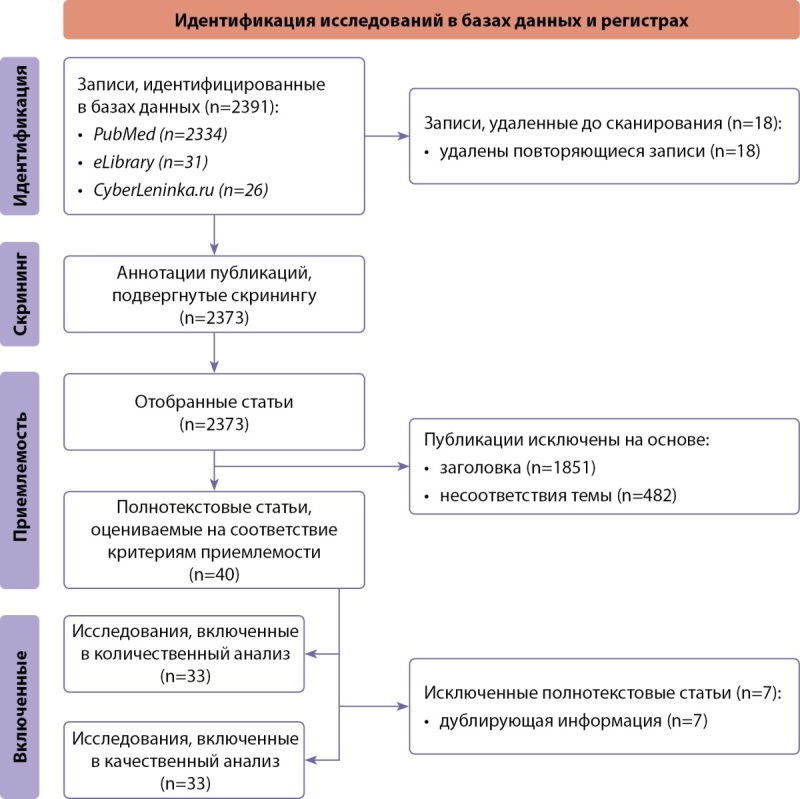
Рисунок 1. Блок-схема дизайна проведенного исследования.Примечание: блок-схема выполнена авторами (согласно рекомендациям PRISMA).

## Характеристика исследований, включенных в анализ

Исследования включали в общей сложности 36 898 пациенток. Типы исследований: девятнадцать проспективных [8][9][17][19][21–23][25][26][28][31][32][36][39][41][42][44][46][47], шесть ретроспективных [11][15][16][33][34][40], шесть перекрестных [6][7][10][27][37][45] и одно пилотное [48].

Риск смещения: не оценивался.

Обобщенные результаты

Клиническая характеристика пациенток с функциональной гипоталамической аменореей и синдромом поликистозных яичников

Как известно, этиологические факторы описываемых заболеваний разные. ФГА является следствием выраженного психоэмоциального напряжения, профессиональных занятий спортом или нездорового стремления к снижению веса. В исследованиях было обнаружено, что депрессия и расстройства пищевого поведения (РПП) являются наиболее распространенными психологическими нарушениями у больных данной эндокринопатией [5]. У женщин с гипоталамической аменореей депрессивные эпизоды наблюдались значительно чаще, чем у здоровых участниц исследований [6]. Однако в некоторых работах было подчеркнуто, что у пациенток с ФГА регистрируют симптомы дисфории, не соответствующие критериям клинического диагноза депрессивного расстройства [7][8].

Частота РПП у пациенток с ФГА неуклонно растет, но в большинстве случаев диагноз не регистрируется в медицинской документации. В исследовании A. Tranoulis почти у половины больных, по данным анкетирования, имелись нарушения пищевого поведения, такие как жесткое соблюдение диеты и склонность к перееданию [9]. Что касается пациенток с СПЯ, в своей работе С.T. Tay и соавт. выявили, что РПП, особенно булимия, чаще встречались при гиперандрогении (11% участниц), чем в контрольной группе (7,6%) [10]. Крупнейшее шведское исследование на предмет изучения психопатологии у женщин с СПЯ включало 24 385 пациенток. В основной группе был повышен показатель отношения шансов (ОШ) наличия хотя бы одного психического расстройства (OШ=1,56 [ 95% доверительный интервал (ДИ), 1,51–1,61]). Наличие гиперандрогении увеличивало риск развития булимии (ОШ 1,21), но снижало риск нервной анорексии (ОШ 0,72). Пациентки с СПЯ оказались в группе риска по развитию булимии, шизофрении, биполярного, депрессивного и тревожного расстройств и расстройств личности [11]. Примечательно, что женщины с ФГА и СПЯ могут страдать булимией одинаково часто, но ограничительный режим питания чаще связан с гипоталамической аменореей. Стоит отметить, что распространенность расстройств питания намного выше у профессиональных спортсменов (18–31%) по сравнению с населением в целом (5–9%) [12]. Более того, существует термин «триада спортсменок», обусловленный гипоэстрогенией и включающий аменорею, РПП и МПКТ. Таким образом, психическая составляющая является пусковым механизмом формирования ФГА, что не всегда характерно для органической патологии — СПЯ. Ответ на вопрос, являются ли первичными или вторичными психопатологические процессы при овариальной гиперандрогении, требует проведения дальнейших рандомизированных исследований [13].

Существуют специфические особенности нарушений менструального цикла изучаемых нозологий. Например, у женщин с ФГА обычно наблюдается аменорея, тогда как при СПЯ чаще встречается олигоменорея. Данное расстройство характеризуется увеличением длительности менструального цикла и частотой менструаций менее девяти в год, тогда как аменорея — это стойкое отсутствие циклического кровотечения в течение трех месяцев при ранее регулярном цикле или в течение шести при нерегулярном. СПЯ диагностируют у 80–90% женщин с олигоменореей и у 40% пациенток с аменореей [14].

Еще одним важным отличием овариальной гиперандрогении и гипоталамической аменореи являются антропометрические характеристики больных. Пациентки с ФГА имеют значительно более низкий индекс массы тела (ИМТ) по сравнению с женщинами с СПЯ: 20,1±2,9 кг/м² и 31,1±7,8 кг/м² соответственно (р<0,001) [15]. ИМТ можно рассматривать как параметр для дифференциальной диагностики, но он должен соотноситься с другими анамнестическими и клиническими данными, а как самостоятельный критерий использоваться не может.

Клиническая (гирсутизм, алопеция, акне) или биохимическая гиперандрогения является ключевым диагностическим критерием СПЯ. Распространенность гирсутизма при классическом фенотипе (сочетание всех трех роттердамских критериев) достигает 75%. Его традиционно оценивают по шкале Ферримана-Галлвея (>4–6 баллов в зависимости от этнической принадлежности). Распространенность алопеции оценивается по визуальной шкале Людвига, общепринятой визуальной шкалы оценки акне не существует [16]. При этом пациентки с ФГА могут также предъявлять жалобы на акне, например, в подростковый период или алопецию. Патологическое выпадение волос, вероятно, обусловлено общим дефицитом микронутриентов, недостаточной калорийностью и отсутствием разнообразия продуктов с учетом ограничительного питания и РПП [17][18].

Лабораторная и инструментальная диагностика СПЯ и ФГА

Дифференциальную диагностику СПЯ и ФГА следует начинать с подробного сбора анамнеза. Как было описано выше, информация о стрессе, физических нагрузках является очень важной, так как уже на начальном этапе может сориентировать врача в нужном направлении. Однако бывает не все так однозначно. Например, пациентки не всегда воспринимают ежедневный стресс для организма (тяжелая учеба, регулярные тренировки) и не придают этому серьезное значение. Немаловажную роль в развитии ФГА играет индивидуальная реакция на стресс и предрасположенность к тревожным расстройствам.

Надо признать, что патогенез заболеваний существенно отличается. Ключевым пунктом в механизме развития считают особенности патологической нейроэндокринной регуляции. При ФГА происходит подавление всей оси гипоталамус-гипофиз-яичники, при этом пульсация гипоталамического ГнРГ снижена, что далее приводит к угнетению выработки гонадотропинов — фолликулостимулирующего гормона (ФСГ), ЛГ гипофизом и в итоге — к гипоэстрогении. СПЯ, напротив, характеризуется увеличением пульсационных выбросов ГнРГ. Концентрации гонадотропинов у пациенток с ФГА ожидаемо будут низкие, особенно ЛГ, в отличие от больных СПЯ [19]. Поэтому именно уровень ЛГ, отражающий изменения пульсации ГнРГ, является значимым маркером в дифференциальной диагностике ФГА, в отличие от ФСГ.

Нейроны, секретирующие кисспептин, нейрокинин В и динорфин (KNDy-нейроны), регулируют импульсную активность ГнРГ-нейронов [20]. Если предположить, что уровни циркулирующего кисспептина отражают работу гипоталамических нейронов, можно было бы ожидать, что показатель кисспептина будет повышен при СПЯ и снижен при ФГА. В исследовании A. Podfigurna и соавт. концентрации кисспептина соответствовали импульсам ЛГ у женщин с ФГА и были ниже при величине ЛГ≤3 МЕ/л [ 1,7 нг/мл] по сравнению с участницами с показателем ЛГ>3 МЕ/л [ 2,6 нг/мл] [21]. В 2016 г. Т. Hofmann и соавт. изучили клинико-лабораторные данные пациенток с нервной анорексией и выявили отрицательную корреляцию концентрации данного гипоталамического нейропептида с интенсивностью физических упражнений (r=-0,41, p=0,01) и положительную — с ИМТ (r=0,514, p<0,001). В этом исследовании также было отмечено, что внутривенное введение кисспептина восстанавливало нормальную пульсационную секрецию ЛГ и ФСГ у больных ФГА [22].

Нейрокинин B (NKB) принадлежит к семейству белков тахикининов, регуляция которых необходима для адекватного функционирования репродуктивной системы. Поскольку секреция кисспептина регулируется передачей сигналов NKB, можно предположить, что у пациенток с ФГА также будет аномальная секреция NKB. В результате проведенного исследования, в котором приняли участие 147 больных ФГА и 88 здоровых женщин, сопоставимых по возрасту, были обнаружены более низкие средние уровни NKB в сыворотке крови в основной группе по сравнению с контролем: 628,35±324,92 и 721,41±337,57 нг/л соответственно (p= 0,002) [23].

Патогенез СПЯ, как и при ФГА, непосредственно связан с нарушениями нейроэндокринной регуляции. Однако при этом есть существенные различия. Во-первых, в случае СПЯ эти расстройства носят органический характер, а во-вторых, при овариальной гиперандрогении усилена импульсная секреция ГнРГ. Работа последнего регулируется не только половыми стероидами по механизму отрицательной обратной связи, но и множеством нейропептидов и нейротрансмиттеров. D. Panidis и соавт. были первыми, кто сравнил уровни кисспептина в сыворотке крови у пациенток с СПЯ и здоровых женщин. Они обнаружили, что данный показатель отрицательно коррелирует с ИМТ, индексом свободных андрогенов и индексом инсулинорезистентности (HOMA-IR). J. Liu и соавт. на основе большого метаанализа (1282 участницы: 699 пациенток с СПЯ и 583 женщины контрольной группы), показавшего повышенные значения этого нейропептида в основной группе по сравнению с контрольной, предложили гипотезу, согласно которой высокий уровень кисспептина можно рассматривать как независимый маркер синдрома [24].

В исследовании Li Wang и соавт. оценивались биохимические и гормональные показатели при СПЯ. В группе пациенток с гиперандрогенией выявлены более высокие уровни липидов в крови, ЛГ и антимюллерова гормона (АМГ), чем у женщин контрольной группы. Изменения метаболизма глюкозы и липидов, а также гормональные нарушения в основной группе, как с ожирением, так и без него, были более значимыми. Результаты ROC-анализа продемонстрировали высокую прогностическую точность объединенных показателей для диагностики СПЯ: AMГ+количество антральных фолликулов (AUC=0,913) и AMГ+ЛГ (AUC=0,901) [25]. На основании этих работ показатель АМГ теперь рассматривают в качестве дополнительного маркера овариальной гиперандрогении. Средние уровни этого гормона в клетках гранулезы яичников in vitro у пациенток с ановуляторным фенотипом СПЯ в 75 раз выше по сравнению с женщинами с регулярной овуляцией [26]. При СПЯ и морбидном ожирении (ИМТ>40 кг/м²) уровень АМГ в сыворотке крови значительно выше по сравнению с контрольной группой, сопоставимой по весу без СПЯ [27]. Однако в другом исследовании у женщин с ожирением и СПЯ уровень АМГ был ниже, чем в группе с СПЯ и нормальным ИМТ [28].

Одним из критериев диагностики СПЯ является гиперандрогения [29]. Хотя и существуют фенотипы этого заболевания без повышения выработки мужских половых гормонов, преобладающая часть пациенток имеют биохимическую и/или клиническую гиперандрогению. Стоит отметить, что для постановки диагноза необходимо исследовать уровень общего тестостерона и глобулина, связывающего половые гормоны (ГСПГ), с последующим расчетом индекса свободных андрогенов. Более предпочтителен метод тандемной масс-спектрометрии [30]. Однако с учетом его ограниченной доступности и высокой стоимости определение уровня тестостерона проводится иммуноферментными методами. Осложняет дифференциальную диагностику тот факт, что гиперандрогения встречается и у пациенток с ФГА. Отличие состоит в том, что избыток андрогенов при гипоталамической аменорее является следствием активации оси гипоталамус-гипофиз-надпочечники, как защитный механизм при различных видах стресса. Другой возможной причиной стоит рассматривать сосуществование двух патологий, когда СПЯ «задавлен» и может реализоваться при устранении стрессового фактора.

Например, в работе J.G. Wang и соавт. было обнаружено, что у женщин с ФГА и поликистозными яичниками (ПКЯ) по данным УЗИ повышена вероятность развития гиперандрогении после стимуляции гонадотропинами, а при увеличении ИМТ на 5–18% у пациенток с ФГА развивались олигоменорея и гиперандрогения [31]. В исследовании V. Mattle и соавт. у 6 из 120 больных ФГА манифестировали признаки СПЯ после пульс-терапии ГнРГ [32]. A. Dumont и соавт. сравнили показатели 40 женщин с ФГА и ПКЯ по данным УЗИ и 27 пациенток с гипоталамической аменореей и нормальной структурой яичников. Авторы не обнаружили различий в уровнях андрогенов и гонадотропинов, а ответ на пульсирующую терапию ГнРГ в итоге значимо не отличался [33].

Примечательно исследование K. Beitl и соавт. о пациентках с неандрогенным фенотипом СПЯ (n=58) и с ФГА и поликистозной морфологией яичников (n=58), сопоставимых по возрасту и ИМТ (ретроспективное исследование «случай-контроль»). В 1-й группе были обнаружены значительно более высокие уровни ЛГ, эстрадиола, тестостерона и более высокое соотношение ЛГ/ФСГ, а также более низкие уровни ГСПГ по сравнению со 2-й группой (p<0,05). Далее были рассчитаны пороговые значения для прогнозирования ФГА-ПКЯ с помощью индекса Юдена. Самая высокая чувствительность была обнаружена для уровня эстрадиола в сыворотке крови менее 37,5 пг/мл — 84,5% (95% ДИ: 72,6–92,6), тогда как отношение ЛГ/ФСГ менее 0,96 имело самую высокую специфичность — 94,8% (95% ДИ: 85,6–98,9). Линейный дискриминантный анализ концентрации тестостерона, ГСПГ и ЛГ позволил правильно классифицировать пациенток с ФГА-ПКЯ в 87,9% случаев (95% ДИ: 80,2–94,0%) [34]. Другие исследования показали, что изменения, характерные для СПЯ (повышенный уровень АМГ и ПКЯ по данным УЗИ), могут быть случайной находкой почти у 40% женщин с ФГА, а у 10% может быть сопутствующая овариальная гиперандрогения [35]. Таким образом, у когорты женщин с ФГА и низким ИМТ при увеличении веса и изменении образа жизни может проявиться и доминировать СПЯ.

Инсулинорезистентность является важнейшим патогенетическим звеном овариальной гиперандрогении. В связи с этим в дифференциальной диагностике следует использовать и методы, способные продемонстрировать нарушение чувствительности к инсулину у пациенток. Одним из наиболее доступных способов является определение индекса HOMA-IR (глюкоза натощак (ммоль/л) х инсулин натощак (мкЕд/мл) / 22,5). Данный метод был описан R.S. Legro и соавт. HOMA-IR отражает чувствительность к инсулину у женщин с СПЯ и ожирением и обладает как высокой чувствительностью, так и специфичностью для выявления инсулинорезистентности [36].

В исследовании Haolin Zhang и соавт. оценивали взаимосвязь композиционного состава тела и инсулинорезистентности у пациенток с гиперандрогенией. У женщин с СПЯ наблюдалась более низкая чувствительность к инсулину, чем в популяции, при увеличении процента жировой ткани в организме. Уровни ЛГ, общего тестостерона, андростендиона и соотношение ЛГ/ФСГ были значительно выше у больных СПЯ по сравнению с контрольной группой, независимо от показателей отношения окружности бедер к окружности талии и объема жировой ткани. Была отмечена отрицательная корреляция между показателями жирового обмена (процент жировой ткани и ИМТ) и гормональным профилем (ЛГ, андростендион) в группе пациенток с СПЯ в отличие от контрольной группы [37]. Таким образом, при овариальной гиперандрогении может присутствовать инсулинорезистентность независимо от наличия избыточного веса. Учитывая повышенный риск развития нарушений углеводного обмена у женщин с СПЯ, целесообразно проведение глюкозотолерантного теста. Это исследование рекомендовано всем пациенткам как наиболее точный способ оценки гликемического статуса, независимо от ИМТ [38]. При этом для пациенток с ФГА характерны сниженные показатели инсулина [39].

Поликистозная структура яичников является одним из критериев СПЯ. Морфологически яичники имеют центральную строму, окруженную периферически расположенными фолликулами, тогда как увеличенное количество фолликулов в яичнике без этого типичного периферического распределения фолликулов было описано как мультифолликулярная структура яичников. При этом у женщин с ФГА встречается увеличение количества фолликулов в яичниках, что затрудняет дифференциальную диагностику [40]. При проведении УЗИ следует обращать внимание на структуру: при СПЯ более характерно распределение фолликулов по периферии, тогда как при ФГА может встречаться мультифолликулярное строение с распределением фолликулов по всему объему яичников.

Клинические особенности пациенток с ФГА обусловлены последствиями гипоэстрогении, такими как снижение МПКТ и повышение риска кардиальной патологии вследствие потери протективного действия эстрогенов на костную и сердечно-сосудистую системы. По данным различных авторов, остеопения регистрируется у 25–90%, а остеопороз — у 19–44% взрослых женщин с нервной анорексией [41]. У женщин с аменореей МПКТ снижается на 2,4% в бедре и на 2,6% в позвоночнике ежегодно [42]. При активных физических нагрузках, которые привели к ФГА, плотность костной ткани тоже низкая, хотя и в меньшей степени, чем у пациенток с нервной анорексией. Z-критерий <-2,0 и от -1,0 до -2,0 были зарегистрированы у 15,4 и 39,8% спортсменок соответственно [43]. Профессиональный спорт может запускать не только ФГА, но и нарушать микроархитектуру и прочность костей, а также увеличивать риск стрессовых переломов (28–47% участниц), при этом сохраненный менструальный цикл эти риски частично нивелирует (17–25,6% участниц) [44][45].

Если ФГА манифестирует в молодом возрасте, это необратимо ухудшает прирост костной массы, поскольку ее пик достигается к 18–25 годам. У женщин с началом заболевания до 18 лет снижение МПКТ нижних отделов позвоночника развивается быстрее, независимо от продолжительности аменореи. Кроме того, может быть нарушен конечный рост костей [46]. Несмотря на увеличение веса и восстановление менструального цикла, больные, у которых наблюдается потеря костной массы в подростковом возрасте, имеют ее хронический дефицит и повышенный риск переломов во взрослом возрасте [47].

Интересны работы, в которых оценивались особенности костной ткани и риск переломов у женщин с СПЯ [48]. Оказалось, что у пациенток с овариальной гиперандрогенией риск переломов ниже, чем у здоровых женщин, и, возможно, это объясняет, почему данное заболевание сохраняет свою высокую распространенность в популяции, несмотря на негативные репродуктивные последствия.

Риск предвзятости по всем исследованиям: не оценивался.

Дополнительные анализы: дополнительных анализов для данного исследования не было предусмотрено.

## ОБСУЖДЕНИЕ

## Интерпретация результатов

В статье были проанализированы возможные способы дифференциальной диагностики ФГА и СПЯ. Мы отметили, что в опубликованных работах особую роль отводится первоначальному сбору анамнеза — по всем канонам диагностического поиска являющимся первичным звеном в дальнейшей цепи действий врача-клинициста. Таким образом, опрос пациенток на предмет наличия стрессовых ситуаций или нарушений питания, как в сторону ограничения и снижения калорийности, так и наоборот склонности к перееданию, должен являться неотъемлемой частью амбулаторного приема докторов. Немаловажным является характер нарушений менструального цикла: для ФГА более характерен длительный период отсутствия менструаций, тогда как при СПЯ чаще встречается олигоменорея. Необходимо выявлять и оценивать выраженность клинических проявлений гиперандрогении и наличие симптомов гипоэстрогении. В данном обзоре проанализированы публикации, посвященные лабораторной и инструментальной диагностике данных патологий как вспомогательных методов не только в установлении диагноза, но и для обнаружения осложнений. Исследования нейроэндокринной регуляции репродуктивной системы способствуют созданию новых способов выявления заболеваний. По мнению ряда авторов, изменения уровней нейропептидов (NKB и кисспептин) можно использовать как маркеры вышеописанных патологий. В представленной работе подробно освещена тема нарушений МПКТ у пациенток с длительным анамнезом аменореи, как одного из важнейших инвалидизирующих последствий гипоэстрогении.

Ограничения исследования: в данном исследовании в силу его особенностей, обусловленных анализом и разработкой критериев дифференциальной диагностики, ограничения не могут быть обсуждены.

## Значение результатов

Данный обзор посвящен социально-значимым патологиям, так как нарушения в работе репродуктивной системы неизбежно приводят к снижению фертильности женщин, особенно при длительном течении и отсутствии лечения. Проведенная в этом исследовании оценка методов дифференциальной диагностики ФГА и СПЯ поможет своевременно выявлять данные заболевания, проводить адекватную медикаментозную терапию и при необходимости подключать к лечению врачей психиатрического профиля с целью коррекции нарушений пищевого поведения и проведения когнитивно-поведенческой терапии.

## ЗАКЛЮЧЕНИЕ

Несмотря на то, что ФГА и СПЯ имеют контрастную патофизиологию, на практике дифференцировать эти две распространенные причины менструальных нарушений бывает непросто. В литературе существуют большие различия в критериях, используемых для определения как СПЯ, так и ФГА.

Особую важность в диагностическом поиске представляет подробный сбор анамнеза. Наличие психоэмоционального стресса или нарушения режима питания характерны для пациенток с ФГА.

Фундаментальное патофизиологическое различие между данными нозологиями заключается в изменении секреции ГнРГ. При СПЯ частота и амплитуда выбросов ГнРГ повышены, а ФГА характеризуется выраженным снижением выработки ГнРГ, что приводит к патологическим изменениям секреции ЛГ, ФСГ. Низкие значения гонадотропинов, особенно ЛГ (<3 МЕ/л), свидетельствуют в пользу гипоталамической аменореи.

В этом обзоре освещены особенности диагностики ФГА и СПЯ, включающие расчет ИМТ, определение показателей ЛГ, андрогенов, инсулина, АМГ и ГСПГ, оценку МПКТ. Проанализированы относительно новые способы выявления заболеваний, такие как измерение кисспептина и NKB. Представленные данные о сходствах и различиях данных патологий могут способствовать своевременной и правильной постановке диагноза.

## ДОПОЛНИТЕЛЬНАЯ ИНФОРМАЦИЯ

Источники финансирования. Работа выполнена в рамках государственного задания №123021300169-4 «Эпигенетические предикторы и метаболомная составляющая аменореи различного генеза у женщин репродуктивного возраста», 2023–2025 гг.

Конфликт интересов. Авторы декларируют отсутствие явных и потенциальных конфликтов интересов, связанных с содержанием настоящей статьи.

Участие авторов. Все авторы одобрили финальную версию статьи перед публикацией, выразили согласие нести ответственность за все аспекты работы, подразумевающую надлежащее изучение и решение вопросов, связанных с точностью или добросовестностью любой части работы.
